# JNK in Tumor Microenvironment: Present Findings and Challenges in Clinical Translation

**DOI:** 10.3390/cancers13092196

**Published:** 2021-05-03

**Authors:** Shing Yau Tam, Helen Ka-Wai Law

**Affiliations:** Department of Health Technology and Informatics, Faculty of Health and Social Sciences, The Hong Kong Polytechnic University, Kowloon, Hong Kong; helen.law@polyu.edu.hk

**Keywords:** cancer stem cells, inflammation, JNK, stressful conditions, tumor microenvironment

## Abstract

**Simple Summary:**

Stress-activated c-Jun N-terminal kinases (JNKs) are members of mitogen-activated protein kinases (MAPKs). Apart from having both tumor promoting and tumor suppressing roles in cancers due to its impact on apoptosis and autophagy pathways, JNK also plays complex roles in the heterogeneous tumor microenvironment (TME) and is involved in different tumorigenesis pathways. The JNK pathway influences various stressful and chronic inflammatory conditions along with different cell populations in TME. In this review, we aim to present the current knowledge of JNK-mediated processes in TME and the challenges in clinical translation.

**Abstract:**

The c-Jun N-terminal kinases (JNKs) are a group of mitogen-activated protein kinases (MAPKs). JNK is mainly activated under stressful conditions or by inflammatory cytokines and has multiple downstream targets for mediating cell proliferation, differentiation, survival, apoptosis, and immune responses. JNK has been demonstrated to have both tumor promoting and tumor suppressing roles in different cancers depending on the focused pathway in each study. JNK also plays complex roles in the heterogeneous tumor microenvironment (TME). JNK is involved in different tumorigenesis pathways. TME closely relates with tumor development and consists of various stressful and chronic inflammatory conditions along with different cell populations, in which the JNK pathway may have various mediating roles. In this review, we aim to summarize the present knowledge of JNK-mediated processes in TME, including hypoxia, reactive oxygen species, inflammation, immune responses, angiogenesis, as well as the regulation of various cell populations within TME. This review also suggests future research directions for translating JNK modulation in pre-clinical findings to clinical benefits.

## 1. Introduction

**Figure 1:**
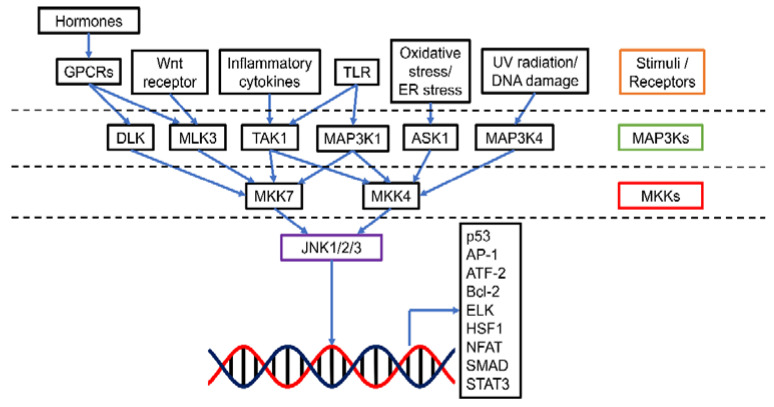
Schematic presentation of the JNK pathway. JNK could be activated by a range of stimuli via specific MAP3Ks. MAP3Ks in turn mediate MKK4 and MKK7 (MKKs). The activated MKKs then up-regulate JNK1/2/3 and allow the transcription of various downstream targets for tumorigenesis events such as apoptosis, cell proliferation, survival, differentiation, inflammation, migration, and metastasis. Abbreviations (other than those defined in the main text): GPCR, G-protein-coupled receptor; ATF-2, Activating transcription factor 2; ELK, E26 transformation-specific like protein; HSF1, Heat shock factor 1; NFAT, Nuclear factor of activated T-cells; SMAD, Mothers against decapentaplegic homolog; STAT3, Signal transducer and activator of transcription 3.

The c-Jun N-terminal kinases (JNKs), also known as stress-activated protein kinases (SAPKs), are an important category of mitogen-activated protein kinases (MAPKs) (Figure 1). There are 10 isoforms of JNKs encoded by 3 genes, namely *jnk1*, *jnk2*, and *jnk3*. While JNK1 (Mapk8) and JNK2 (Mapk9) expression is ubiquitous in most tissues, JNK3 (Mapk10) is mainly expressed in brain, heart, and testes [1]. Compared with JNK1 and JNK2, JNK3 has an extended NH_2_-terminal region [2]. The isoforms are expressed as both the short form (46 kDa for JNK1 and JNK2, 48 kDa for JNK3) and the long form (55 kDa for JNK1 and JNK2, 57 kDa for JNK3) [2]. The 10 splice variants of JNKs have a high level of homology (80%) yet there are some differences in the COOH-terminal region and alternative exons within the protein kinase domain, leading to the binding selectivity of JNK [2,3]. Similar to other MAPKs, activation of JNK requires dual phosphorylation at tyrosine (Tyr185) and threonine (Thr183) residues by two upstream regulators MAPK kinase (MKK), 4 (MKK4) and MKK7. MKK7 is a specific activator of JNK while MKK4 can also activate p38 MAPK [4]. MKK4 and MKK7 can be activated by a number of MKK kinases (MAP3Ks), including transforming growth factor-β-activated kinase 1 (TAK1/MAP3K7) and apoptosis signal-regulating kinase 1 (ASK1/MAP3K5). TAK1 activates JNK via inflammatory cytokines (interleukin-1 (IL-1), lymphotoxin-b, transforming growth factor-β (TGF-β), tumor necrosis factor-α (TNF-α)), and toll receptors (TLR-3, 4 and 9) [1,5]. Additionally, TAK1 could transduce signals from various receptor systems in both the innate and adaptive immunity [6]. ASK1 responds to oxidative stress and endoplasmic reticulum stress [7]. Other MAP3Ks, including MAP3K1, MAP3K4, dual leucine zipper kinase (DLK), and mixed-lineage kinase 3 (MLK3), can also activate JNK when induced by stresses such as ultraviolet irradiation, DNA damage, toll receptors, and hormones [8].

Downstream, JNK mediates cell proliferation, differentiation, survival, apoptosis, and immune responses [9]. In this regard, activator protein-1 (AP1) composed of Fos and Jun members is the major JNK target. Phosphorylated JNK dimerizes Jun proteins, particularly c-Jun with Fos proteins (c-Fos, FosB, Fra-1, and Fra-2) to form AP-1 [1]. AP-1 then participates in cell proliferation, survival, differentiation, inflammation, migration, and metastasis [9]. Other downstream targets of JNK include the members of mitochondrial apoptosis regulator Bcl-2 family (Bcl-2, Bcl-xl, Bad, Bim, and Bax) and tumor suppressor p53, leading to the pro-apoptotic function of JNK [1,10].

Regarding the role of JNK in cancer, previously JNK has been conceived as an apoptosis driver for cell death, thus it could act as a tumor suppressor as demonstrated in breast cancer [11], oral cancer [1], and pancreatic cancer [12]. However, recent reports suggested that JNK is also an autophagy promoter for cell survival under stressful conditions including low oxygen level by removing dysfunctional cellular components [13,14,15,16]. In addition, JNK could be activated by proinflammatory cytokines, growth factors and immune cells favoring tumor development [4,6,17]. In addition, JNK could be an important mediator of various steps in metastasis, ranging from epithelial–mesenchymal transition (EMT) to growth in secondary sites [18].

**Figure 2:**
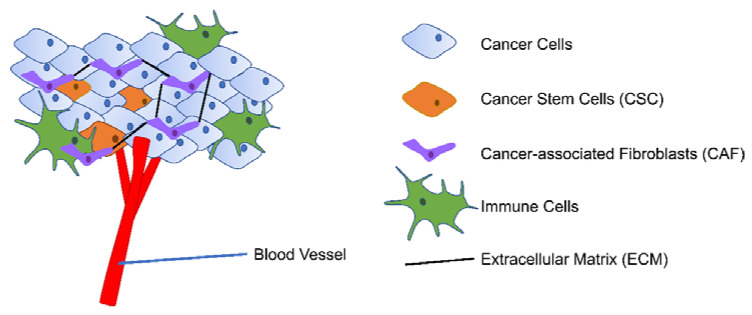
Simplified presentation of TME. TME consists of proliferating tumor cells with CSC population, blood vessels, stromal cells (mainly CAF), immune cells, and ECM.

The tumor microenvironment (TME) consists of proliferating tumor cells with specific cancer stem cell (CSC) populations, blood and lymphatic endothelial cells, stromal cells (mainly cancer-associated fibroblasts (CAF)), immune cells, and extracellular matrix (ECM) Figure 2 [19,20]. Adding to the complexity of the cellular components, the heterogeneous TME also has varied levels of oxygen and pH and different degree of inflammation [21,22]. TME is closely linked with tumor development along with prognosis and cancer treatment efficacy by enabling immune escape, angiogenesis, and extravasation to the bloodstream [22,23]. As cancer is one of the leading causes of death worldwide, further understanding of the complexity of TME is thus rewarding in improving cancer treatment outcomes [24].

**Figure 3:**
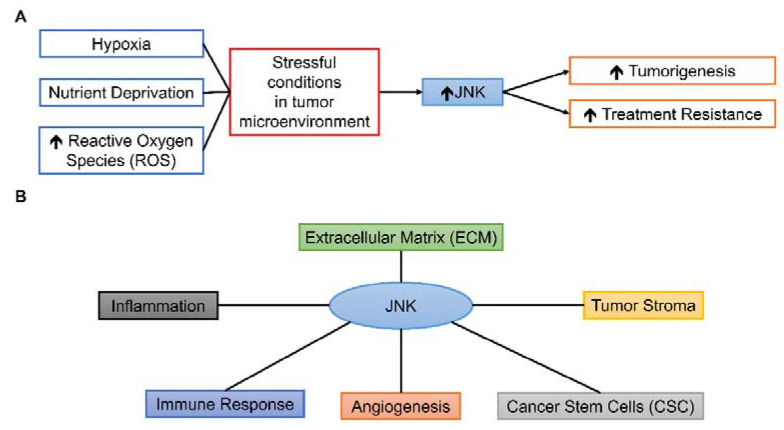
Possible roles of JNK in TME. (**A**) Various stressful conditions can activate the JNK pathway, leading to tumorigenesis and treatment resistance. (**B**) JNK can influence various components of TME, including inflammation, immune response, angiogenesis, CSC, tumor stroma, and ECM.

While JNK could have prominent roles in various components of TME due to its responsiveness to different stimuli and wide-ranged cellular effects Figure 3, the roles of JNK in TME are complex and under extensive research. This review aims to summarize the present findings of JNK-mediated processes in various TME conditions and components, drawing from different types of human cancer Table 1 and to suggest possible future research directions in translating pre-clinical findings of JNK to clinical settings.

## 2. Relationship of JNK with Stressful Conditions in TME

Hypoxia is a common phenomenon in most solid tumors due to abnormal vascularization and poor blood supply despite angiogenesis promotion by tumor cells [21,54]. Although hypoxia inducible factors (HIFs) have been widely accepted as the major pathways involved in hypoxic response, the JNK pathway also plays an important role in hypoxia-induced tumorigenesis processes [54]. Vasilevskaya et al. [25] demonstrated hypoxia-induced JNK relates to the treatment responses of colorectal cancer (CRC) cells to DNA damaging agents including oxaliplatin. The administration of JNK inhibitor CC-401 could sensitize several CRC cell lines to chemotherapy both in vitro and in vivo. The research team further found that JNK1 inhibition could attenuate hypoxia-induced autophagy and increase chemotherapy treatment efficacy of the CRC cell line HT-29 [15]. Our team also demonstrated sequential autophagy key regulation activation with JNK acting as the delayed autophagy promotor under both hypoxia (1%) and blood oxygen level (10%) in HT-29 cells [14]. We subsequently discovered that the JNK pathway mediates low oxygen level induced EMT and stemness maintenance in CRC cell lines HT-29, DLD-1, and SW-480 [26]. The addition of specific JNK inhibitor SP600125 could achieve partial relief of EMT induction and stemness maintenance under hypoxia and blood oxygen level. These observations collectively show that JNK participates in different hypoxia-induced tumorigenesis events and treatment resistance in CRC. Apart from CRC, Zhou et al. [27] demonstrated hypoxia-induced invasion of lung adenocarcinoma cell line A549 could be inhibited by the specific JNK inhibitor SP600125 due to the suppression of c-Jun expression.

Due to the overpopulation of tumor cells and restricted access to nutrients, nutrient depletion is also a stress factor in TME [21]. JNK could also be activated by nutrient-depletion as it responds to a wide range of stresses [1,7]. Roca et al. [28] evaluated the role of the JNK pathway in the PC3 prostate cancer cell line under nutrient-depletion stress. They showed that JNK activation mediated IL-4-induced proliferation under nutrient-depletion stress while JNK inhibition could stunt IL-4-induced cell proliferation.

High reactive oxygen species (ROS) level is another common feature of many solid tumors [55]. ROS consist of superoxide, hydrogen peroxide, and hydroxyl radicals. In tumor cells, ROS are mainly produced by metabolic reactions occurring within the mitochondria. They may contribute to oxidative damage to DNA, leading to genomic instability [55]. ROS accumulation has been linked with JNK activation as ROS could inhibit MAP kinase phosphatases, thus preventing inactivation of JNK [56]. Another link has been suggested with the mediation of ASK1-JNK for redox control of ROS [57]. However, the exact role of JNK in ROS homeostasis within tumor cells has been rarely investigated. Li et al. [29] studied the role of the JNK pathway in ROS-induced cell death among CRC, hepatocellular carcinoma (HCC), and cervical cancer cells. They concluded that JNK-associated leucine zipper protein (JLP)-JNK pathway was activated by hydrogen peroxide. It also protected tumor cells from ROS-induced necrosis. These researchers demonstrated the roles of JNK in a range of tumorigenesis events influenced by different stressful conditions, though further studies are warranted to consolidate the actual roles of JNK in different cancer types under various stressful conditions.

## 3. Relationship of JNK with Inflammation in TME

Chronic inflammation is often a hallmark of TME and mainly activated by oncogenes including receptor tyrosine kinases (RTKs), Ras and p53 mutant [22,23]. Ras initiation promotes pro-malignant paracrine mechanism mainly by IL-6 and IL-8 [58]. Ras and p53 mutants together promote NF-κB inflammatory pathway [23]. The major features of inflammation in TME consist of the presence of pro-inflammatory cytokines including IL-1 family and TNF-α, chemokines such as C-C motif ligand 2 (CCL2) and IL-8/CXCL8, immune responses, and angiogenesis [59]. JNK takes important roles in these inflammation-related events in the TME as supported by the following evidence.

### 3.1. JNK and Pro-Inflammatory Cytokines/Chemokines in TME

Pro-inflammatory cytokines can up-regulate JNK through TAK1 [1]. JNK has also been concluded to have a key role in Ras-initiated tumor formation in lung tissue of mice [60]. Many JNK downstream targets also modulate the activation of inflammatory mediators such as E-selectin, IL-2, matrix metalloproteinases, and TNF-α [61]. Das et al. [30] showed the complicated relationship between JNK and HCC development. JNK could promote an inflammatory hepatic environment to support tumor development by regulating IL-1, IL-6, TGF-β, and TNF-α. However, JNK deficiency in hepatocytes could in turn promote HCC initiation. Han et al. [31] further demonstrated that JNK mediated myeloid cells in hepatitis and HCC development. JNK was involved in hepatic inflammation by up-regulating inflammatory cytokines (interferon γ (IFN-γ), IL-1, IL-6, and TNF-α) and chemokines (CCL2 and CCL5). Takahashi et al. [32] reported that JNK1 mediated inflammation in tobacco smoke-promoted lung tumorigenesis by promoting IL-6 and TNF-α. A close relationship between JNK and IL-6-induced cell migration was also demonstrated in oral squamous cell carcinoma [33].

IL-33 is a relatively new member of the IL-1 family and it is expressed by non-hematopoietic cells such as endothelial cells within human tumor tissues of kidney, stomach, liver, pancreas, lung, breast, or colon cancers [62]. IL-33 binds and activates its receptor IL-1 receptor-like 1 (IL1RL1/ST2) and is associated with inflammation and cancer progression [34]. Recent studies have linked IL-33 with the JNK pathway in several cancer types. Wu et al. [34] found that IL-33 promoted renal cell carcinoma tumor growth and chemotherapy resistance by the JNK pathway. Tong et al. [35] echoed the close relationship between IL-33 and JNK in ovarian cancer cell proliferation and metastasis. Similar findings were also reported in gastric cancer as JNK inhibition blocked the chemotherapy resistance by IL-33 [36]. IL-33 could also promote CRC stemness by activating core stem cell genes via the JNK pathway [37]. Additionally, this research also demonstrated that IL-33 could recruit macrophages into TME and stimulate them to produce prostaglandin E_2_ that supported CRC stemness and tumor growth.

### 3.2. JNK and Immune Responses in TME

Immune cells are important members of TME due to the presence of chronic inflammation. They are generally categorized as tumor promoting (Th2 cells, regulatory T cells (Treg), M2-like macrophages, myeloid-derived suppressor cells) and tumor suppressing (Th1 cells, cytotoxic T lymphocytes (CTL), dendritic cells, and M1 macrophages) [63]. Tumor-associated macrophages (TAM) are considered a major component of leukocytic infiltration in TME of most solid tumors [38]. They inhibit lymphocyte (CTL and Th1 cells) functions by producing inhibitory cytokines like IL-10, prostaglandins, or ROS [20]. For innate immune response, close connection between JNK and TAM was found. Hagemann et al. [38] showed JNK promoted macrophage-induced invasiveness in breast and ovarian epithelial cancer cells. Further investigation found that JNK mediated macrophage activity via extracellular matrix metalloprotease inducer (EMMPRIN) and macrophage migration inhibitory factor (MIF) expressions in tumor cells. Another study reported that the JNK pathway participated in intermittent hypoxia-induced pro-inflammatory phenotype in M0 and M1 macrophages [64]. For adaptive immune response, JNK is required for helper T cell proliferation and effector cell functioning [65]. The study of different JNK isoforms reveals that JNK1 deficiency could result in hypo-proliferative CTL with lower IL-2 production while JNK2 deficiency could conversely result in hyper-proliferation of CTL [66]. JNK also reduced the proliferative response of activated Th cells and potentiated polarized T cell differentiation into the Th1 lineage [67].

Apart from directly influencing immune cells in TME, JNK could also participate in immune evasion of tumor cells in TME. Immune evasion by tumor cells is a series of manipulations by tumor cells to avoid tumor eradication by immunity and promote tumor development [68]. The complex mechanisms of immune evasion are linked to the heterogeneous tumor microenvironment, which maintains itself as an immunosuppressive environment for tumor development [69].

JNK could influence immune evasion of tumor cells by manipulating T lymphocytes. The JNK signaling pathway was involved in TGF-β-induced invasion and metastasis of gastric carcinoma [70], while JNK also modulated TGF-β activation in liver carcinoma cells [71]. TGF-β is an immunosuppressive cytokine that suppresses CTL response by IFN-γ inhibition and Treg cell promotion [72]. TGF-β could also suppress T cell proliferation by inhibiting IL-2. In CRC metastasis, TGF-β took an important role in immune evasion [73] and the increased TGF-β level predicted poor prognosis in CRC patients [74]. These observations suggested that JNK could influence immune evasion by modulating TGF-β production.

Another possible downstream target for JNK-induced immune evasion is via the programmed cell death protein 1 (PD-1)/ programmed death-ligand 1 (PD-L1) pathway. PD-1 was up-regulated on T cell activation and the level remained high on exhausted T cells [75]. Its binding with the ligand PD-L1 on tumor cells led to inhibition of T cell activation and suppression of effector T cell responses [75]. Moreover, the up-regulation of PD-L1 expression in tumor cells could be seen as an effective means of CTL suppression [75]. JNK could up-regulate PD-L1 through TLR4 signaling in bladder cancer cells [39] and through nitric oxide in glioblastoma cells [40], indicating the possible JNK-induced PD-L1 expression among tumor cells for immune evasion.

Apart from TGF-β and PD-L1 mediation by JNK, JNK is an up-stream regulator of protein kinase B (Akt) [26], which could be another prominent effector of immune evasion. In a study of lung tumor cell immune evasion, Noh et al. [76] found that the activation of the Akt pathway enabled immune evasion from CTL-mediated apoptosis. Further studies linked Akt activation with the regulation of PD-L1 and showed that Akt inhibition could improve CTL antitumor effects in breast, prostate, skin, and pancreatic cancers [77]. Conclusively, JNK activation in tumor cells could actively enable immune evasion by multiple effectors.

## 4. JNK and Angiogenesis/Endothelial Cells in TME

Angiogenesis is essential for tumor development, especially when the tumor is greater than 1–2 mm in diameter. Thus, endothelial cells are indispensable components in the TME [78]. Stressful conditions and chronic inflammation in TME contribute to the high angiogenic capacity of tumor tissues. However, tumor vessels are abnormal in structure and thus produce a vicious cycle of creating a hypoxic and acidic TME [79]. JNK is indispensable in angiogenesis. JNK1 increased *vegf* mRNA expression by binding c-Jun to its promoter and JNK3 promoted endothelial cell migration for angiogenesis [18]. Uchida et al. [80] further showed that JNK mediated Egr-1 for proliferation and migration, and matrix metalloproteinase-2 (MMP-2) and membrane type-1 (MT1)-MMP for proteolysis in endothelial cells. Another study linked JNK with cyclooxygenase-2 (COX-2) in vascular endothelial growth factor (VEGF)-induced angiogenesis in endothelial cells [81]. These reports signify the prominent role of JNK within endothelial cells on angiogenesis. Apart from angiogenesis mediation, the JNK pathway also facilitated E-selectin expression in endothelial cells for promoting adhesion of CRC cells to endothelial cells and trans-endothelial migration [41]. These processes contributed to the extravasation of circulating tumor cells and eventual metastasis.

The JNK activation in tumor cells is correlated with angiogenesis in several cancer types. JNK activation promoted VEGF-A, CXCL1, CXCL5, IL-8/CXCL8, and MMP-1 through inflammatory cytokine IL-1α in human gastric cancer cell lines [42]. Similar angiogenesis induction by JNK in head and neck squamous cell carcinoma [43] and ovarian cancer [44] was also established. Along the same line, Yang et al. [45] reported JNK mediated TGF-β1-induced angiogenesis in an improved zebrafish embryo/xenograft glioma model. The group further concluded that p38 MAPK, extracellular signal-regulated kinase (ERK), and phosphoinositide 3-kinase (PI3K) did not participate in the process. Together, these reports firmly established the importance of the JNK pathway in mediating angiogenesis within tumor cells.

## 5. JNK and CSC in TME

CSC are transformed tumor cells that have different population sizes in many solid tumors. The definition of CSC is constantly changing with new research findings, and it is beyond the scope of this review [19]. Basically, tumors consist of a mixture of self-replicating CSC, non-replicating tumorigenic cells, and cells of intermediate state, contributing to the heterogenous tumor concept [82]. CSC retain self-renewal and differentiation capacities, i.e., stem cell-like, and contribute to treatment resistance [19]. The treatment resistance mechanisms initiated include angiogenesis, EMT, immune escape, and resistance to hypoxia. In turn, stressful conditions and other cell populations within TME also have great influence on CSC progression and stemness of tumors [19].

Roles of JNK for CSC subpopulation and stemness maintenance of a variety of cancer types were evaluated with conflicting conclusions. Okada et al. [46] reported that the JNK inhibitor AS602801 could decrease the viability, self-renewal, and tumor-initiating capacity of CSC in pancreatic cancer, non-small cell lung cancer, ovarian cancer, and glioblastoma in vitro. Additionally, systemic administration of AS602801 in xenograft tumors could reduce CSC population in vivo. Reports from the same research group found JNK inhibition by pharmacological drugs or genetic targeting could result in the loss of self-renewal and tumor-initiating capacity of CSC derived from ovarian cancer cell line A2780 [47] and stem-like glioblastoma cells [48]. This research group further demonstrated that the use of K-Ras knockdown [83] or dexamethasone-induced MKP-1 for inactivating JNK [84] in pancreatic cancer. Modulation of K-Ras or MKP-1 subsequently reduced CSC burden in pancreatic cancer both in vitro and in vivo. The connections of JNK activation and stemness maintenance in CRC were also established by the low oxygen level induced [26] and IL-33-induced [37] JNK activation. Our group found that low oxygen level induced JNK activation could promote stemness maintenance in CSC cells by up-regulating stemness markers octamer-binding transcription factor 4 (Oct4) and NANOG [26]. Liu et al. [85] also concluded that JNK is essential for maintaining stemness and tumor-initiating ability in chemoresistant human cancer cells, whereas Xie et al. [49] found that knockdown of JNK1 or JNK2 or the addition of pan-JNK inhibitor JNK-IN-8 could reduce ALDH1^+^ and CD44^+^/CD24^−^ CSC subpopulations, and mammosphere formation in triple-negative breast cancer. They also demonstrated that the JNK-c-Jun pathway promoted CSC phenotype by NOTCH1 signaling and administration of JNK inhibitor, JNK-IN-8, in a xenograft model could suppress tumor growth by inhibiting CSC phenotype acquisition. Insua-Rodríguez et al. [50] also concluded that JNK activation was critical for tumor initiation and metastasis in a xenograft model of breast cancer. Conversely, Girnius et al. [51] found that JNK could prevent tumor initiation and has no role in CSC activity in murine breast cancer. Indeed, the roles of JNK in CSC development may vary between different cancer types and stages with involvement of different stemness signaling pathways. Focused studies to evaluate the roles of JNK isoform will be required to understand the actual roles of the JNK pathway in CSC development.

## 6. JNK and Tumor Stroma in TME

Tumor stroma forms the backbone and supportive structure of TME for the functional epithelial cells, which could constitute up to 80% of the entire tumor [21,86]. CAFs are the major constituent of tumor stroma with differed properties from fibroblasts in normal tissue [87]. The recruitment and activation of CAFs are mainly controlled by growth factors from tumor cells and immune cells including TGF-β, inflammatory modulators, platelet-derived growth factor (PDGF), and fibroblast growth factor 2 (FGF2) [87,88]. CAF can influence tumor growth by exerting metabolic effects, immune crosstalk with T cell function, secretion of soluble factors for macrophage manipulation, angiogenesis, and cancer invasion, along with ECM remodeling [88]. CAF can even influence treatment resistance by diverse mechanisms including alteration in cell-matrix interactions, cell-cell interactions, formation of physical barriers, and activation of epigenetic plasticity in neighboring cells [86,88].

There are few studies conducted on the role of the JNK pathway in tumor stroma or ECM. Lisanti et al. [89] found that the JNK1 pathway was up-regulated in tumor stroma of breast cancer patient samples, while Sato et al. [52] indicated JNK1 inhibition in tumor stroma of pancreatic ductal adenocarcinoma had potential treatment effects. Their results suggested that CAF with JNK activation would enhance expression of chemokine Ccl20 and cause reduction of CTL infiltration, and JNK1 knockout in a murine model could reduce this effect. Wang et al. [53] studied the role of CAF in cisplatin resistance of lung cancer cells. They found the connection between annexin A3 (ANXA3) expression in CAF with cisplatin resistance via the JNK pathway as JNK inhibition could suppress the effect of ANXA3 on cisplatin sensitivity. For the effect of the JNK pathway on ECM, a breast cancer study showed that the JNK pathway could increase the expressions of ECM proteins secreted phosphoprotein 1 (SPP1) and tenascin C (TNC) [50]. The JNK-induced SPP1 and TNC also promoted lung metastasis and chemotherapy resistance. These reports provide hints that JNK may have roles in tumor stroma and ECM development.

## 7. Challenges in Clinical Translation of JNK Modulation

The JNK pathway is mediated by a range of upstream effectors, and can influence a variety of downstream targets in tumorigenesis. The cellular conditions and components of TME have been considered as major players in tumor progression, with heterogeneous stressful conditions and complicated interactions between different cell types [20,21]. The development of specific therapeutic strategies targeting components of TME should be able to improve treatment outcomes [22]. As demonstrated from the presented evidence, the JNK pathway has prominent roles in TME progression. Nevertheless, there are relatively few clinical trials conducted on JNK inhibitors. There are various difficulties for translating the pre-clinical knowledge of the JNK pathway in TME to clinical benefits. First, actions of JNK on cancer development are complex. JNK could have both tumor promoting and tumor suppressing roles depending on tumor type and developmental stage. For example, conflicting reports on JNK-mediated CSC development and tumor initiation of breast cancer are available [49,50,51]. This may be due to different tumor type investigated and thus deeper investigation of the exact intrinsic or extrinsic factors contributing to the diverse results is suggested. Development of frameworks for stratification of positively responding patients is required to improve the efficacy of clinical translation. Second, the roles of different isoforms of JNK contributing to tumorigenesis and TME development are still not well investigated. Most of the presented studies employ pan-JNK inhibitors such as SP600125 instead of specific JNK isoform inhibitors. These inhibitors lack specificity and thus the specific roles of JNK1, JNK2, and JNK3 in TME are not well defined yet [90,91]. There are some JNK isoform-specific inhibitors being developed such as AV-7 (JNK1 inhibitor) [92]. Yet further development of specific JNK isoform inhibitors is warranted for better understanding of JNK isoform-specific TME-related events. Third, the current JNK inhibitors are systemic drugs which affect both normal and malignant tissues with noticeable adverse effects [93]. There was limited success in using JNK inhibitors in clinical trials with most trials discontinued by sponsors due to lack of efficacy and unpredicted side effects [94]. Targeted drug delivery deep into the necrotic tumor core and specific cell population of TME could be a possible way for clinical translation to reduce incidence of adverse effects [22,95,96]. Nanomedicine-based approach offers a promising drug delivery method as nanoparticles can be functionalized with different moieties for targeting specific cell population within TME [22]. Currently, preclinical and clinical trials targeting immune response, angiogenesis, and remodel matrix of TME are under progress [96]. The use of nanoparticle may potentially translate the JNK modulating treatment strategy for clinical benefits.

## 8. Conclusions

In summary, the JNK pathway could have great influence in tumor progression in TME as it can be activated by stresses and inflammatory cytokines, and mediate the immune responses, endothelial cells, CSC, and stromal cells. Inhibition of the JNK pathway could generally improve treatment outcomes or suppress tumor progression in TME. However, studies on the JNK pathway are usually less intensive than other more ‘famous’ mediators such as HIFs for hypoxic responses, NF-κB for inflammatory responses, VEGF for angiogenesis, and TGF-β for immune responses and CAF development. Additionally, multiple isoforms of JNK could have both pro-oncogenic and anti-oncogenic roles identified depending on cancer type and stage. Therefore, in-depth research on JNK isoform-specific TME development events and methods for stratifying responsive patients are warranted to exploit JNK modulation as a novel cancer treatment approach in clinical settings.

## Figures and Tables

**Table 1:** Overview of JNK-mediated processes in TME conditions and components.

TME Condition/Component Involved	Cancer Type(s)	Role of the JNK Pathway	Reference
Hypoxia	CRC	↑ Autophagy	[14]
		↑ Autophagy, chemotherapy resistance	[15]
		↑ Chemotherapy resistance	[25]
		↑ EMT, stemness maintenance	[26]
	LC	↑ Invasion	[27]
	PRC	↑ Proliferation	[28]
Nutrient Depletion	CEC, CRC, HCC,	↓ Necrosis	[29]
High ROS	HCC	↑ Inflammatory cytokines	[30]
Inflammation	HCC	↑ Inflammatory cytokines and chemokines	[31]
	LC	↑ Inflammatory cytokines	[32]
	ORC	↑ Migration	[33]
	RC	↑ Tumor growth, chemotherapy resistance	[34]
	OVC	↑ Proliferation, metastasis	[35]
	GC	↑ Chemotherapy resistance	[36]
	CRC	↑ Stemness, tumor growth	[37]
	BRC, OVC	↑ TAM-induced invasiveness	[38]
Immune Response	BLC	↑ PD-L1	[39]
	G	↑ PD-L1	[40]
	CRC	↑ Adhesion to endothelial cells, trans-endothelial migration	[41]
Angiogenesis/ Endothelial Cells	GC	↑ Angiogenesis, inflammatory cytokines	[42]
	HNC	↑ Angiogenesis	[43]
	OVC	↑ Angiogenesis	[44]
	G	↑ Angiogenesis	[45]
CSC	CRC	↑ EMT, stemness maintenance	[26]
		↑ Stemness, tumor growth	[37]
	G, LC, OVC, PAC	↑ Viability, self-renewal, tumor-initiating capacity of CSC	[46]
	OVC	↑ Self-renewal, tumor-initiating capacity of CSC	[47]
	G	↑ Self-renewal, tumor-initiating capacity of CSC	[48]
	BRC	↑ CSC phenotype, tumor growth	[49]
		↑ Tumor initiation, metastasis	[50]
		↓ Tumor initiation	[51]
Tumor Stroma	PAC	↑ Ccl20 in CAF causing ↓ CTL infiltration	[52]
	LC	↑ ANXA3 in CAF causing ↓ chemotherapy sensitivity	[53]
ECM	BRC	↑ SPP1, TNC causing ↑ lung metastasis, chemotherapy resistance	[50]

Abbreviations: Cancer type: BLC, Bladder Cancer; BRC, Breast Cancer; CEC, Cervical Cancer; CRC, Colorectal Cancer; G, Glioblastoma/ Glioma; GC, Gastric Cancer; HCC, Hepatocellular Carcinoma; HNC; Head and Neck Cancer; LC, Lung Cancer; ORC, Oral Cancer; OVC, Ovarian Cancer; PAC, Pancreatic Cancer; PRC, Prostate Cancer; RC, Renal Cancer.
